# Acute bilateral ureteropelvic junction obstruction as a rare cause of hypertensive crisis: a case report

**DOI:** 10.1186/s13256-022-03431-6

**Published:** 2022-05-23

**Authors:** Bruce Adrian Casipit, Jerald Pelayo, Joseph Alexander Paguio, Jasper Seth Yao, Neil Shah

**Affiliations:** Department of Medicine, Einstein Medical Center Philadelphia, Philadelphia, PA USA

**Keywords:** Bilateral ureteropelvic junction obstruction, Hypertensive crisis, Nephrolithiasis

## Abstract

**Background:**

Bilateral ureteropelvic junction obstruction is a common cause of secondary hypertension in the pediatric population, often due to congenital malformation. On the other hand, it is less frequently encountered in the adult population and is usually due to an acquired condition, most commonly by a bilaterally obstructing nephrolithiasis causing hydronephrosis and subsequent hypertension. The aim of this study was to investigate and highlight the underlying mechanisms by which acute bilateral ureteropelvic junction obstruction causes hypertensive crisis and why early detection and prompt treatment are necessary to mitigate the effects of elevated blood pressure on target organs.

**Case presentation:**

A 41-year-old African American man with hypertensive cardiomyopathy presented with anuria. He was found to have elevated blood pressure with evidence of target organ damage on laboratory examination, demonstrated by sudden elevation of his serum creatinine level. He was initially treated with oral and intravenous antihypertensives, with minimal improvement. The work-up was unremarkable apart from the imaging finding of acute bilateral ureteropelvic junction obstruction from obstructing nephrolithiasis causing hydronephrosis. Bilateral ureteral stents were placed for decompression, with resolution of the hypertensive crisis and improvement of renal function.

**Conclusion:**

This case highlights the importance of prompt diagnosis and treatment of underlying acute bilateral ureteropelvic junction obstruction to mitigate the deleterious effects of sudden blood pressure elevation on target organs.

## Background

Primary hypertension among adults is a common disease entity encountered in the clinical setting and is usually influenced by multiple factors, including age, lifestyle, comorbidities, genetics, and race. On the other hand, hypertension in a young adult should receive prompt evaluation for possible secondary underlying causes to mitigate the long-term complications of elevated blood pressure. These include, but are not limited to, the investigation of possible renal and vascular pathologies, endocrine issues, or drug side effects [1].

Ureteropelvic junction (UPJ) obstruction, as a cause of secondary hypertension, commonly occurs in children secondary to a congenital malformation [2, 3]. It can usually be diagnosed in utero through routine prenatal ultrasound or may present as recurrent urinary tract infections during childhood [3]. On the other hand, secondary hypertension due to an acquired bilateral obstructive uropathy among adults is less frequently encountered [4]. Prompt recognition and treatment of an obstruction is therefore necessary to resolve hypertension, especially in the setting of acute blood pressure elevation.

## Objective

To determine the underlying mechanisms by which acute bilateral UPJ obstruction causes hypertensive crisis and why early detection and prompt treatment are necessary to mitigate the effects of elevated blood pressure on target organs.

## Case presentation

A 41-year-old African American man was brought to the emergency department for evaluation due to 4-day history of progressive decrease in urine output associated with bilateral flank pain. He denied having fever, shortness of breath, dysuria, hematuria, lower-extremity swelling, skin tightening, painful extremities on cold exposure, fatigue, or recent weight loss.

The patient’s past medical history was significant for hypertension and chronic systolic heart failure (left ventricular ejection fraction 15%). The patient had been admitted a year prior for new-onset acute systolic heart failure due to uncontrolled hypertension that was diagnosed when he was in his early twenties and was never treated. He was discharged with losartan, spironolactone, hydralazine, isosorbide dinitrate, and carvedilol, for which he reported good compliance. His family and social history were noncontributory.

On presentation, he had blood pressure of 262/147 mmHg, heart rate of 102 beats/min, respiratory rate of 18 breaths/min, and oxygen saturation of 97% on ambient air. He was alert and oriented and was not in acute respiratory distress. He had faint bilateral crackles at the lung bases. No jugular vein distension or peripheral edema was noted. His heart rhythm was normal, and there were no murmurs or gallops. He had generalized abdominal tenderness without guarding. The urinary bladder was palpable. No costovertebral angle tenderness was shown. The remainder of the examination was unremarkable.

**Fig. 1:**
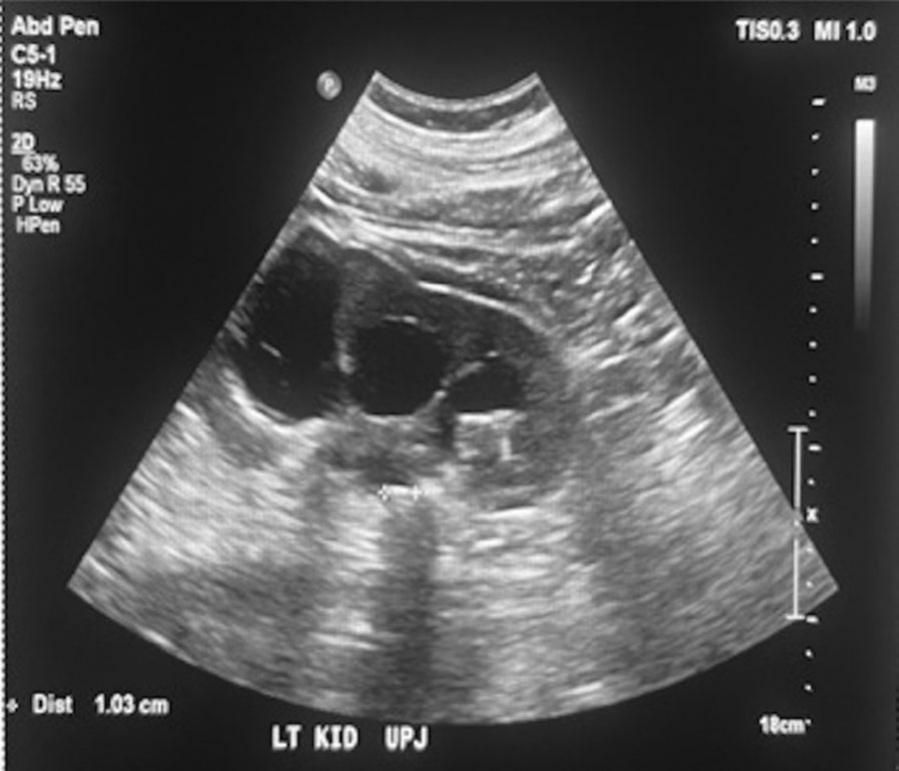
Renal ultrasound showing an obstructive 1-cm calculus at the left UPJ, causing moderate to severe hydronephrosis

**Fig. 2:**
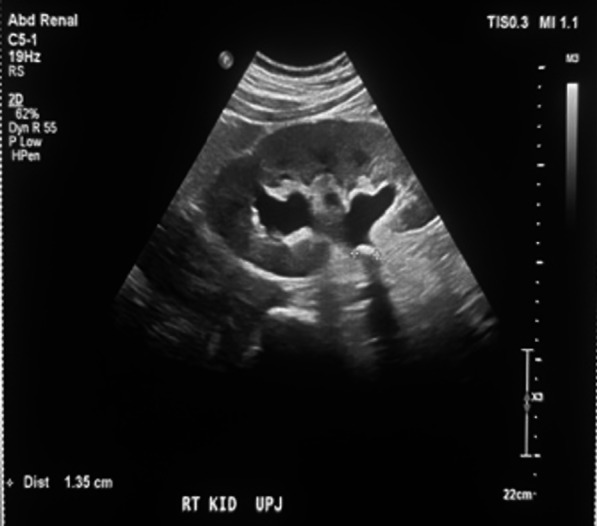
Renal ultrasound showing an obstructive 1.4-cm calculus at the right UPJ, causing moderate hydronephrosis

**Fig. 3:**
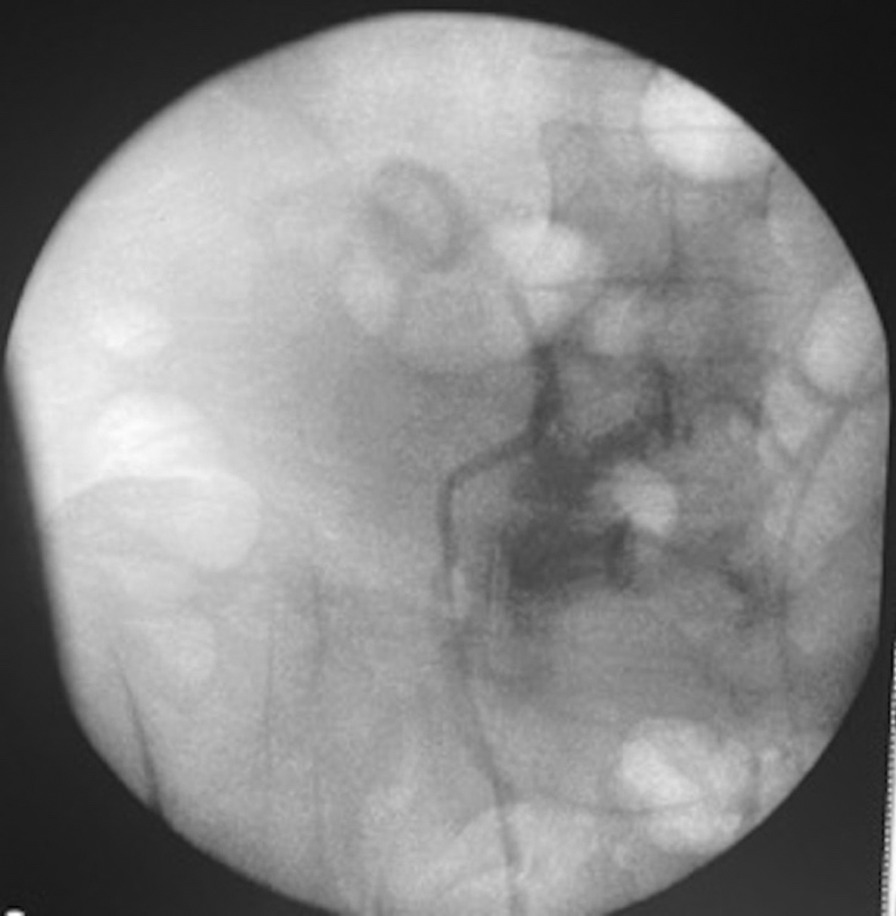
Fluoroscopic retrograde urography showing right renal pelvis dilation with a filling defect consistent with the calculus noted on sonogram

His blood chemistry showed a serum creatinine level of 14.1 mg/dL, increased from his baseline level of 1.6 mg/dL, and a blood urea nitrogen (BUN) level of 57 mg/dL with a BUN-to-creatinine ratio of 4.04. Urinalysis showed microscopic hematuria. Chest radiography revealed mild pulmonary edema. His hemoglobin level was 13 g/dL. He was managed with intravenous labetalol (10 mg), oral carvedilol (25 mg), and oral hydralazine (25 mg), which slightly decreased his high blood pressure by approximately 40 mmHg for his systolic blood pressure (SBP) and 10 mmHg for his diastolic blood pressure (DBP). A straight catheter was inserted, with no urine flow noted. Furthermore, a bladder scan was performed, which showed no urine in the bladder. An ultrasound of the kidneys showed bilateral UPJ obstruction from bilateral obstructing nephrolithiasis measuring 1.4 cm at the right UPJ and 1 cm at the left UPJ, causing moderate right and severe left hydronephrosis (Figs. 1, 2). Furthermore, fluoroscopic retrograde urography showed a dilated right and left renal pelvis with filling defects consistent with calculi noted on ultrasonography (Fig. 3).

**Fig. 4:**
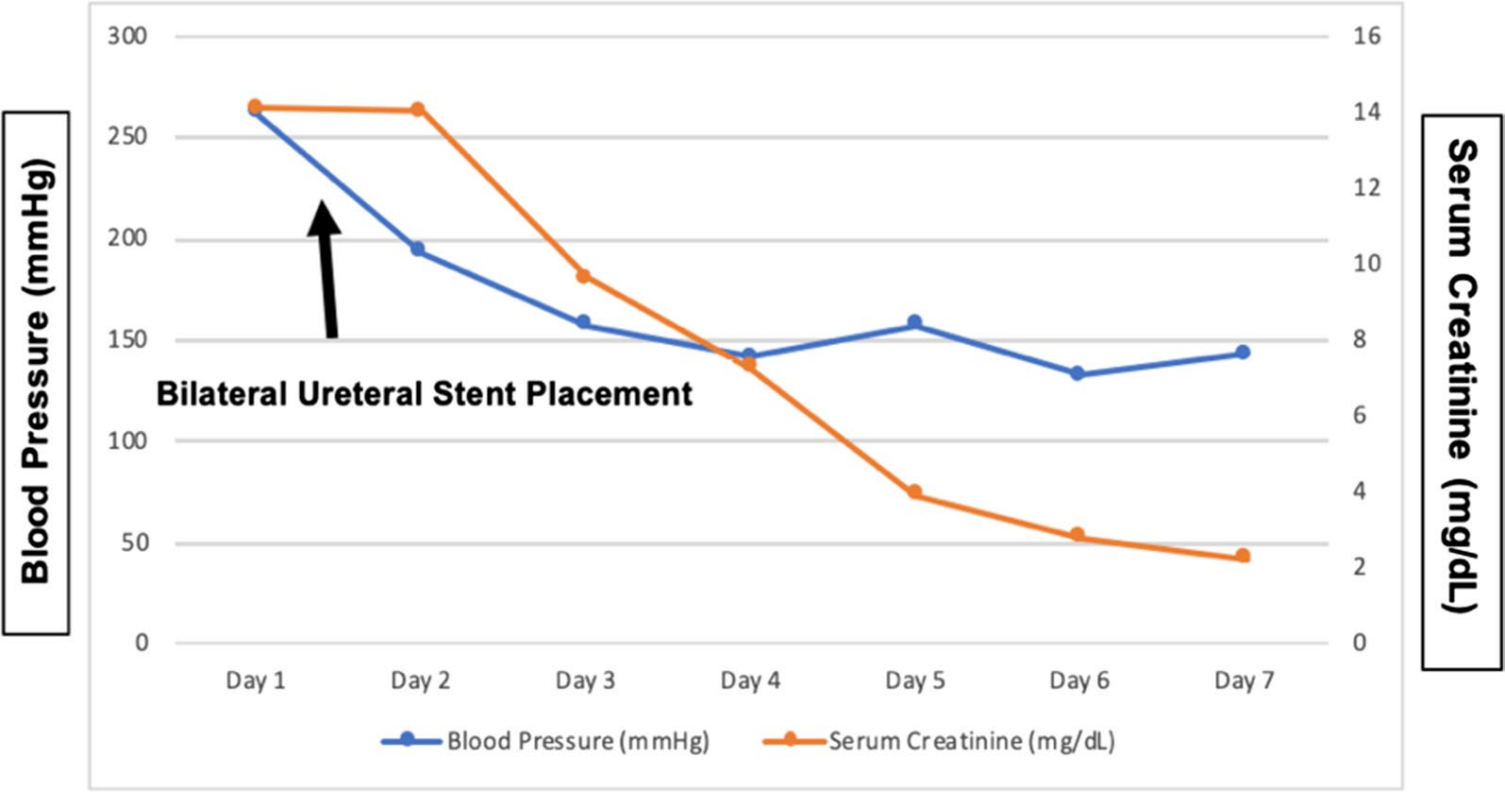
Blood pressure and serum creatinine trends on admission and after bilateral ureteral stent placement

He underwent bilateral retrograde pyelogram, bilateral ureteral stent placement, and cystoscopy. After the procedure, the patient’s blood pressure improved dramatically and returned to his baseline of 140/80 mmHg (Fig. 4). The serum creatinine level also trended downward to 2.2 mg/dL, which was close to his baseline of 1.6 mg/dL (Fig. 4). A Foley catheter was initially placed, and the voiding trial was successful after a few days. Definitive management of bilateral obstructing nephrolithiasis was planned as outpatient treatment.

On follow-up 2 weeks later, the patient reported feeling much better. His office blood pressure was 141/86 mmHg, and his repeat serum creatinine level was 1.8 mg/dL. The urology service then performed a definitive urological intervention to remove the obstructing nephrolithiasis bilaterally with improvement in urine flow.

## Discussion and conclusions

This case illustrates the importance of prompt management of the underlying secondary cause of a hypertensive crisis. In this case, the patient had an acute bilateral UPJ obstruction causing hydronephrosis secondary to bilateral nephrolithiasis.

UPJ obstruction is an anatomical distortion causing the impediment of urinary flow from the renal pelvis to the ureter, increasing back pressure to the kidneys and eventually leading to hydronephrosis. It is most often congenital and is diagnosed during childhood as part of the workup for secondary hypertension [3]. In contrast, acquired UPJ obstruction causing hydronephrosis is more common in adults secondary to nephrolithiasis, previous surgery, or malignancy. Although the incidence of nephroliths causing bilateral obstruction simultaneously is lower, it can still occur and is a plausible cause of secondary hypertensive crisis. Hydronephrosis as a cause of secondary hypertension has been evident in several studies [5–8], although most of these studies described unilateral hydronephrosis. Based on these limited studies, it was concluded that even unilateral obstruction causing hydronephrosis is enough to cause secondary hypertension [5, 7, 8]. On the other hand, there are even limited reports citing acquired bilateral hydronephrosis from bilateral UPJ obstruction as a secondary cause of hypertension. It is postulated that relief of the obstruction causes resolution of the hypertensive crisis. One case report describes rapid resolution of hypertension after bilateral nephrostomy insertion in a patient with bilateral hydronephrosis secondary to a pelvic malignancy. This now leads to the notion that different intrarenal mechanisms play a significant role in causing the elevation of blood pressure in hydronephrotic kidneys [9].

Although the exact mechanisms of hypertension among patients with hydronephrosis are still unknown, two mechanisms are implicated in the development of hypertension in hydronephrotic kidneys. These include increased tubuloglomerular feedback (TGF) activity and activation of the renin–angiotensin–aldosterone system (RAAS) [9].

The RAAS plays a role in the development of hypertension among patients with hydronephrotic kidneys, as evidenced by increased plasma renin activity [9]. In the setting of an obstruction, the urine returns to the renal system, causing stasis and increasing back pressure, leading to hydronephrosis. Although the exact mechanism of increased activity of the RAAS in hydronephrotic kidneys is still unknown, it is postulated that it may be related to reduced renal blood flow because of increased TGF. TGF is a physiological mechanism involving the regulation of glomerular blood flow. With high glomerular flow rates, such as in the setting of volume overload, there is increased sodium delivery to the macula densa, causing release of the afferent arteriolar vasoconstrictor adenosine, leading to decreased glomerular blood flow. Nitric oxide, a modulator of TGF, is downregulated in hydronephrotic kidneys. This causes unrestricted vasoconstriction of the afferent arteriole, decreasing sodium delivery to the macula densa, and thereby activating the RAAS and its downstream effects [9]. The improvement of hypertension after relief of the obstruction suggests that humorally mediated vasoconstriction plays a significant role in the mechanism by which hydronephrosis causes hypertension [9, 10].

The patient in this case developed bilateral nephrolithiasis, which was asymptomatic, eventually causing acute UPJ obstruction and subsequent hydronephrosis. This simultaneous acute bilateral obstruction caused the rapid development of hydronephrosis leading to downstream effects that culminated in a hypertensive crisis. The elevated blood pressure led to target organ damage, including flash pulmonary edema, because of the increased hydrostatic pressure. There was rapid resolution of the elevated blood pressure after relief of the obstruction using bilateral nephrostomy tubes. The patient’s serum creatinine level also greatly improved after relief of the obstruction.

Among patients presenting with acute bilateral UPJ obstruction complicated by a hypertensive crisis, prompt treatment is necessary to ameliorate target end-organ damage. According to the American Urological Association (AUA), an obstructed renal system is a urological emergency, and thus immediate intervention is needed [11]. Recommendations include either ureteric stenting or percutaneous nephrostomy for alleviating the obstruction and have been deemed equally effective in achieving decompression. If sepsis or infection is involved, definitive treatment should be postponed until it is resolved [11, 12]. Although this is true for unilateral ureteral obstruction, the same principle could also be applied for bilateral obstruction. Therefore, regardless of whether the obstruction is unilateral or bilateral, prompt treatment is necessary to decompress the obstruction and alleviate the elevated blood pressure to ameliorate further damage to organs. We report this case of acute bilateral UPJ obstruction due to bilateral obstructing nephrolithiasis causing hypertensive crisis to emphasize the importance of prompt recognition and treatment to mitigate the deleterious effects of elevated blood pressure on target organs.

## Data Availability

Not applicable

## Abbreviations

UPJ: Ureteropelvic junction
RAAS: Renin–angiotensin–aldosterone system
TGF: Tubuloglomerular feedback
