# Correction to: Acute bilateral ureteropelvic junction obstruction as a rare cause of hypertensive crisis: a case report

**DOI:** 10.1186/s13256-022-03499-0

**Published:** 2022-06-29

**Authors:** Bruce Adrian Casipit, Jerald Pelayo, Joseph Alexander Paguio, Jasper Seth Yao, Niel Shah

**Affiliations:** Department of Medicine, Einstein Medical Center Philadelphia, Philadelphia, PA USA

## Correction to: Journal of Medical Case Reports (2022) 16:220 https://doi.org/10.1186/s13256-022-03431-6

Following publication of the original article [1], the authors identified an error in the author name of Niel Shah and changed the wording of a sentence in the the first paragraph of the Background section.

The incorrect author name is: Neil Shah

The correct author name is: Niel Shah

The sentence currently reads: These include, but are not limited to, the investigation of possible renal and vascular pathologies, endocrine issues, or drug side effects [1].

The sentence should read: These include multiple investigations to evaluate for possible renal and vascular pathologies, endocrine issues, or drug side effects [1].

The author group and the wording of a sentence in the first paragraph of the Background section have been updated. The original article [1] has been corrected.
